# Normal Mouse Intestinal Epithelial Cells as a Model for the *in vitro* Invasion of *Trichinella spiralis* Infective Larvae

**DOI:** 10.1371/journal.pone.0027010

**Published:** 2011-10-31

**Authors:** Hui Jun Ren, Jing Cui, Zhong Quan Wang, Ruo Dan Liu

**Affiliations:** Department of Parasitology, Medical College, Zhengzhou University, P. R. China; National Cancer Institute, United States of America

## Abstract

It has been known for many years that *Trichinella spiralis* initiates infection by penetrating the columnar epithelium of the small intestine; however, the mechanisms used by the parasite in the establishment of its intramulticellular niche in the intestine are unknown. Although the previous observations indicated that invasion also occurs *in vitro* when the infective larvae are inoculated onto cultures of intestinal epithelial cells (e.g., human colonic carcinoma cell line Caco-2, HCT-8), a normal readily manipulated *in vitro* model has not been established because of difficulties in the culture of primary intestinal epithelial cells (IECs). In this study, we described a normal intestinal epithelial model in which *T. spiralis* infective larvae were shown to invade the monolayers of normal mouse IECs *in vitro*. The IECs derived from intestinal crypts of fetal mouse small intestine had the ability to proliferate continuously and express specific cytokeratins as well as intestinal functional cell markers. Furthermore, they were susceptible to invasion by *T. spiralis*. When inoculated onto the IEC monolayer, infective larvae penetrated cells and migrated through them, leaving trails of damaged cells heavily loaded with *T. spiralis* larval excretory-secretory (ES) antigens which were recognized by rabbit immune sera on immunofluorescence test. The normal intestinal epithelial model of invasion mimicking the natural environment *in vivo* will help us to further investigate the process as well as the mechanisms by which *T. spiralis* establishes its intestinal niche.

## Introduction

*Trichinella spiralis* is a parasitic nematode that infects their vertebrate host after consumption of meat containing infective larvae. Following their release in the stomach by digestion of meat, the larvae penetrate into host intestinal epithelium where they molt to adulthood, mate, and reproduce the next generation of larvae [1], [2]. The life cycle of *T. spiralis* is completed when newborn larvae invade and mature in striated muscle cells of the new host [3], [4]. During the intestinal phase of infection, the larvae and adult parasites localize to the crypt-villus junction, establishing an intramulticellular niche composed of numerous epithelial cells [5]. The parasites are mobile in the epithelium, continually invading and occupying the cytoplasm of new cells, and do not appear to cross the basement membrane [6].

It is well known that the invasion of host intestinal epithelium by the infective larvae is the first step during *T. spiralis* infections. However, until now the mechanisms by which *T. spiralis* infective larvae recognize, invade, migrate within the intestinal epithelium and establish its intramulticellular niche have not ever been elucidated. The reason why those studies have been hindered lies in the lack of a manipulated *in vitro* model. Previous attempts have showed that when *T. spiralis* infective larvae are suspended in semisolid medium and inoculated onto monolayers of tumor epithelial cells (e.g., human colonic carcinoma cell line Caco-2, HCT-8) grown *in vitro*, they invade cells, penetrate adjacent cells, and reside in the cytoplasm of the syncytia that they create [7], [8], [9]. Obviously, however, the application of human cancer cell lines has been limited by their cancerous nature, especially in mimicking human gut environment and screening the proteins related to invasion by the parasite. Thus, the invasion models will be more natural if normal small intestinal epithelial cells (IECs) can be used, but it is quite difficult to obtain and culture IECs *in vitro*. Previous investigations, using the IEC line derived from rat intestinal crypt epithelium (IEC-6) as an invasion model, have demonstrated that *T. spiralis* infective larvae did not invade IEC-6 cells [10]. Hence, up till now a normal intestinal epithelial model which can reproduce the invasion of small intestinal epithelium by *T. spiralis in vitro* has not been reported.

In this study, small IECs were obtained from intestinal crypts of fetal mouse small intestine. The *in vitro* invasion of mouse IECs by *T. spiralis* infective larvae was determined by microscopic observation. This paper presents for the first time a normal readily invasion model which will be helpful in revealing the mechanism of invasion by *T. spiralis* at the cellular and molecular levels.

## Materials and Methods

### Ethics statement

This study was carried out in strict accordance with the National Guidelines for Experimental Animal Welfare (MOST of People's Republic of China, 2006). All animal procedures reported herein were reviewed and approved by the Zhengzhou University Animal Care and Use Committee (Permission No. SYXK 2007-0009).

### Parasite and bile

The isolate (ISS534) of *T. spiralis* used in the study were obtained from a domestic pig in Nanyang city of Henan Province, China. The isolate was maintained by serial passages in Kunming mice in our laboratory. The muscle larvae were released from the infected mouse muscles by digestion of carcasses with 1% pepsin (1∶3,000) and 1% hydrochloric acid [11]. These mice had been infected at least 42 days prior to collection.

Bile was collected from Kunming mice killed by anaesthetic inhalation with isoflurane (Sigma) and stored at −80°C prior to experimentation. For *in vitro* experiments, the muscle larvae were activated by the mice bile (diluted 1∶20 in saline) at 37°C for 2 h. Then, the larvae were rinsed three times with saline, incubated for 2 h in saline plus antibiotics, and washed on a sieve before being inoculated onto IEC monolayers [7].

### Experimental animals

BALB/c mice aged 6 weeks were purchased from the Experimental Animal Center of Henan province, bred in plastic micro-isolator cages and used for the study.

### Isolation and culture of mouse intestinal epithelial cells

The culture medium used was high-glucose-formulation Dulbecco's modified Eagle's medium (DMEM; Gibco) supplemented with 4 mM glutamine, 20 mM Hepes, 1 mM sodium pyruvate, 100 U/ml penicillin, 100 U/ml streptomycin, 0.1 U/ml bovine insulin (Sigma), and 5% fetal bovine serum (FBS; Gibco), hereafter referred to as the complete DMEM. Cells were grown in 25-cm^2^ plastic culture flasks (Corning).

BALB/c fetuses were removed on embryonic days 17–19 (E17–19) by cesarean section and were kept in ice-cold D-Hank's buffer [12], followed by discarding the mesentery. The small intestines were gently pulled out of the abdominal cavity, opened longitudinally, and immersed in D-Hank's buffer. The intestines were minced into 1 mm long fragments with sharp scissors. The fragment were then incubated at 37°C under agitation for 30 min in the presence of type I collagenase (200 U/ml, Sigma) and hyaluronidase (100 U/ml, Sigma). Incubation solutions were carefully removed and centrifuged at 100× g for 5 min at 4°C. The pellets were washed with DMEM containing 2% FBS and 2% sorbitol, and the supernatant containing the purified crypt fraction was collected by centrifugation at 250× g at room temperature (RT). The crypts were then resuspended and incubated in complete DMEM. In each case, the medium with the non-adhering cells was recovered after 90-min culture and plated into a new plastic culture flask. Medium was changed every 48 h and the confluence reached within approximately 8 days. Subcultures were performed after trypsinization (0.5% trypsin-0.54 mM EDTA in PBS, at 23°C for 5 min).

### Purification of intestinal epithelial cells

In primary culture, fibroblasts were usually mixed with IECs, which may grow either in groups or scattered. As IECs and fibroblasts have different tolerance to trypsin, fibroblasts were detached from the flask wall firstly while the IECs remained attached when they were digested with trypsin. Fibroblasts were then washed away and IECs were left in the flask. The purification of IECs was performed as described previously [13]. In brief, the medium was removed, and the cells were rinsed with D-Hank's buffer twice, followed by digestion with trypsin (2 ml) at 37°C for 2 min. When the fibroblasts contracted, and no changes were observed in IECs, digestion was terminated by the addition of complete DMEM. Fibroblasts were detached with D-Hank's buffer, followed by adding medium. Cell clones were obtained after three passages by screening only a single IEC in each well of 96-well plates where IECs had been diluted to ensure that each well contained only a single cell. Cell populations were expanded after the cloning and transferred to mass culture in cell flasks. The IECs were kept frozen after 8–9 passages in liquid nitrogen.

### Cell proliferation and growth curve

Cell growth was measured by MTT [3-(4,5-diethylthiazoly-2-yl)-2,5-diphenyltetrazolium bromide] assay [14]. Briefly, IECs (passage 8) were harvested conventionally by digestion and seeded at 5,000 cells per well in 96-well culture plates in quadruplicate. At various points in time, medium was removed, and cells were incubated with 20 µl of MTT solution (5 mg/ml; Sigma) at 37°C for 4 h, followed by solubilization with 100 µl of 100% dimethyl sulfoxide (Sigma) at 37°C for 10 min. The absorbance of each well was measured with a microplate reader (Bio-Tek) at a wavelength of 570 nm. The viable cell number is proportional to the absorbance.

### Hematoxylin-and-eosin (HE) and immunofluorescence (IF) staining of IECs

Cells (passage 8) were fixed with ice-cold acetone for 10 min, then hematoxylin-and-eosin (HE) and immunofluorescence (IF) staining were performed. The major steps of IF staining were shown as follows. The cells were incubated with rabbit polyclonal anti-cytokeratin 18 (1∶100; Santa Cruz) at 37°C for 1 h, followed by washing with phosphate-buffered saline (PBS). The cells were then incubated with FITC-conjugated goat anti-rabbit IgG (1∶100; Santa Cruz), and nuclei were stained with propidium iodide (PI). In negative control groups, anti-cytokeratin 18 antibodies were replaced with PBS.

### Invasion and fluorescence assay

IECs were grown to confluence on glass coverslips (in 12-well plates). Each monolayer was overlaid with approximately 120 activated larvae suspended in 0.2 ml of serum-free DMEM containing 15 mM HEPES and 1.75% agarose [7]. The IECs were incubated at 37°C in 5% CO_2_ for 1 h. During incubation, invasion of IECs by *T. spiralis* infective larvae were observed continuously under inverted phase contrast microscope (Olympus). At the end of the experiment, the agarose containing larvae was removed, and the monolayers were stained with trypan blue. The area of damaged cells was quantified by NIH image software (ver. 1.57). A total of 25 fields per monolayer were captured by video microscopy with a 4×objective (Olympus).

After 1 h of co-culture with larvae at 37°C, the monolayers were washed twice with DMEM-10% FBS. The cells were covered with PI (0.03 mg/ml in DMEM-50% FBS) and incubated at 37°C for 2 min, and then at 4°C for 30 min. After being washed twice with DMEM-10% FBS, the cells were fixed in 4% paraformaldehyde for 30 min, washed in PBS, and permeabilized and blocked in blocking solution (PBS containing 0.1% Triton X-100 and 5% normal goat serum) at RT for 20 min. The monolayers were incubated with rabbit immune sera against *T. spiralis* excretory-secretory (ES) antigens (1∶100) at 37°C for 1 h, washed three times in PBS, and followed by 40-min incubation with FITC-conjugated specific secondary antibody (Sigma) at RT in the dark. Subsequently, the coverslips were mounted on glass slides and examined under a fluorescence microscope (Olmpus).

### Statistical analysis

The mean area of stained (damaged or dead) cells per field was estimated for each monolayer, and five monolayers were evaluated per treatment group. Statistical differences between activated groups and non-activated groups were identified by analysis of variance and Scheffés test [7], [9]. The level of significance used was 5% (*p*<0.05).

## Results

### Isolation and culture of mouse intestinal epithelial cells

**Figure 1:**
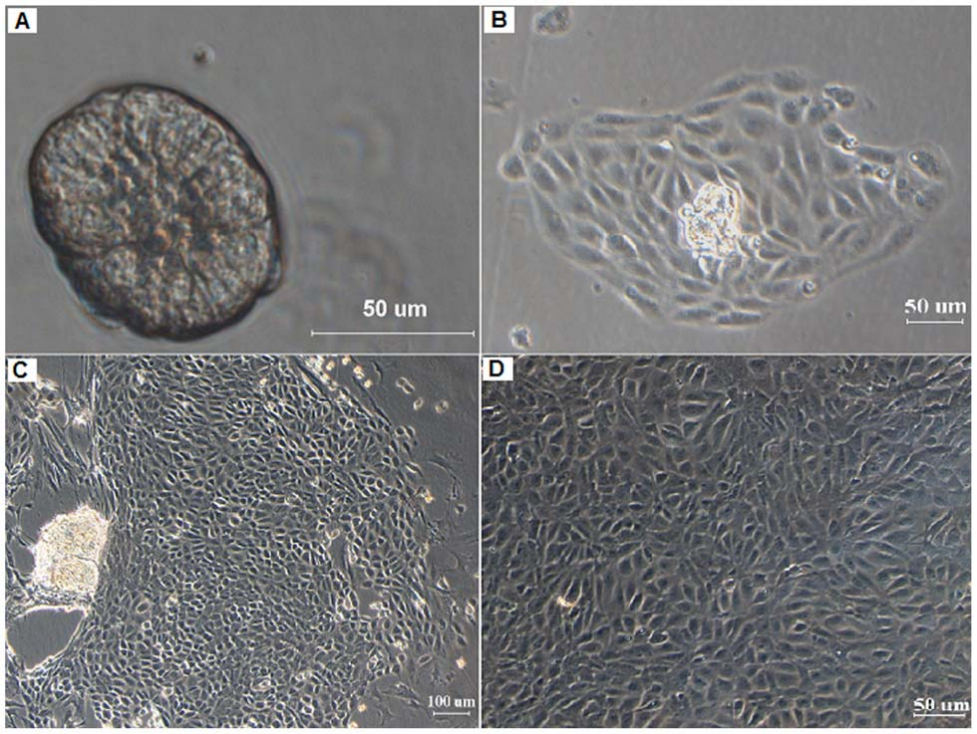
Cultured mouse primary intestinal epithelial cells under an inverted phase-contrast microscope. (A) The single crypts obtained with collagenase-hyaluronidase dissociation of mouse small intestine. (B) A few cells gradually migrated out around the crypts after 24 h. (C) The epithelial cells started to grow rapidly after 48 h, and multiple large colonies were formed at day 5. (D) Cells continued to grow until confluence was reached after 9 days.

After digestion with type I collagenase and hyaluronidase for 30 min, the small intestinal crypts were successfully isolated from tissue samples (Figure 1A). Viability studies using dye exclusion showed that 95% of the cells in the isolated crypts were viable. Most crypts became adherent to the flask within 24 h, and a few cells gradually migrated out around the crypts at 24 h (Figure 1B). Then, the cells continued to divide extensively after culturing for 2 to 9 days (Figure 1C) before they reached confluence (Figure 1D).

### Purification and characterization of cells

Few fibroblasts were observed at passage 3, and then viable IECs were cloned by limiting dilution as described above. The selected cells were maintained in log-phase growth and expanded by transferring sequentially into 24-well plates, 6-well plates and finally T-25 flasks (data not shown).

**Figure 2:**
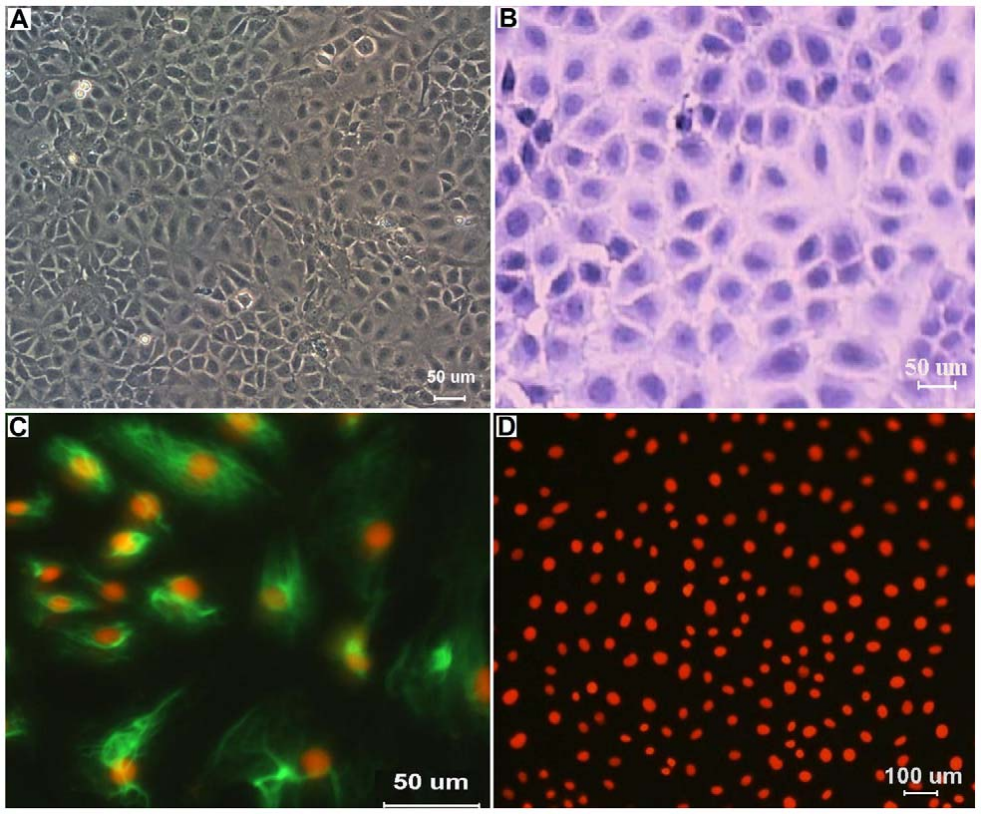
Morphological characteristic and Immunofluorescence staining of fetal mouse IECs. (A) The IECs formed a tightly packed monolayer with typical cobblestone morphology (passage 8). (B) HE staining of IECs. (C) Immunofluorescence staining of IEC cytokeratins, cytokeratin 18 was clearly detected in the cytoplasm of IECs with a green color. (D) No green fluorescence in the cytoplasm of IECs was found and only the nucleus was stained red with propidium iodide (PI) in the negative controls.

Structural characterization of cells was observed under a light microscope. Colonies displayed the characteristic of epithelial cells, such as an adherent monolayer, a characteristic paving-stone-like arrangement and a tightly packed pattern. Each cell had polygonal, flattened shape with a large, oval nucleus, typical features of normal epithelial cells (Figure 2A and 2B). These IECs obtained in our study have been maintained for 17 passages, and no obvious morphological changes have been observed. Cytokeratins in cytoplasm of the IECs were clearly detected by IF, indicating the epithelial characteristics of these cells (Figure 2C). However, no green fluorescence was observed in the negative controls (Figure 2D).

**Figure 3:**
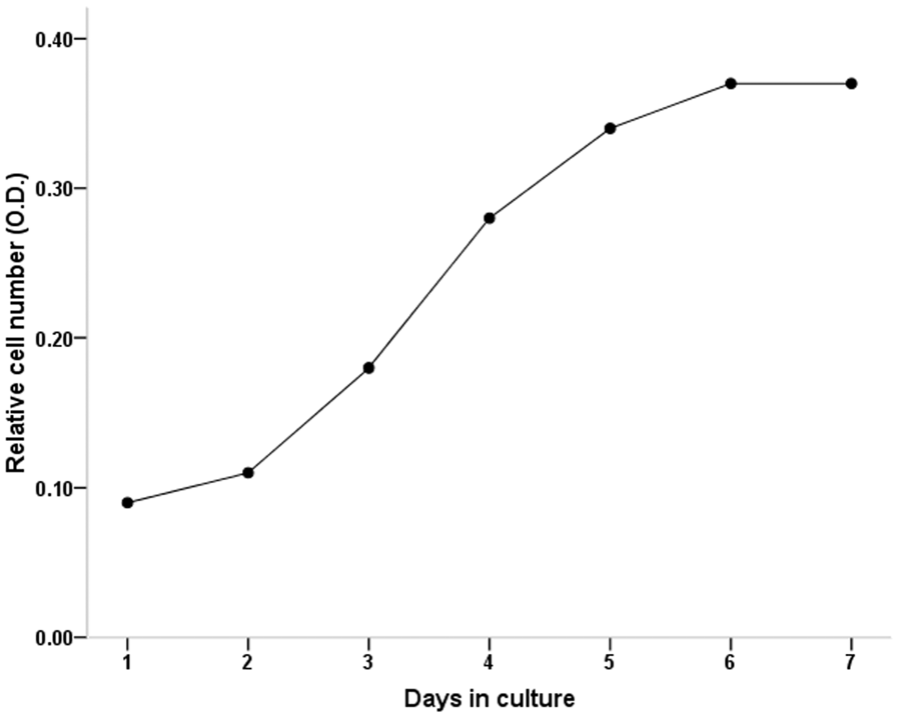
Growth curve of passaged mouse IECs in culture. The log phase started after day 2 of the lag phase with a sharper inclination.

Cell proliferation was measured by the MTT assay, and the growth curve was plotted based on the mean absorbance values of the IECs at the indicated time points (Figure 3). The growth curve showed that after an initial lag of 48 h, the cells entered the log-phase (96 h), and then they stopped growing and reached the plateau phase of growth.

### Invasion and IF assay

**Figure 4:**
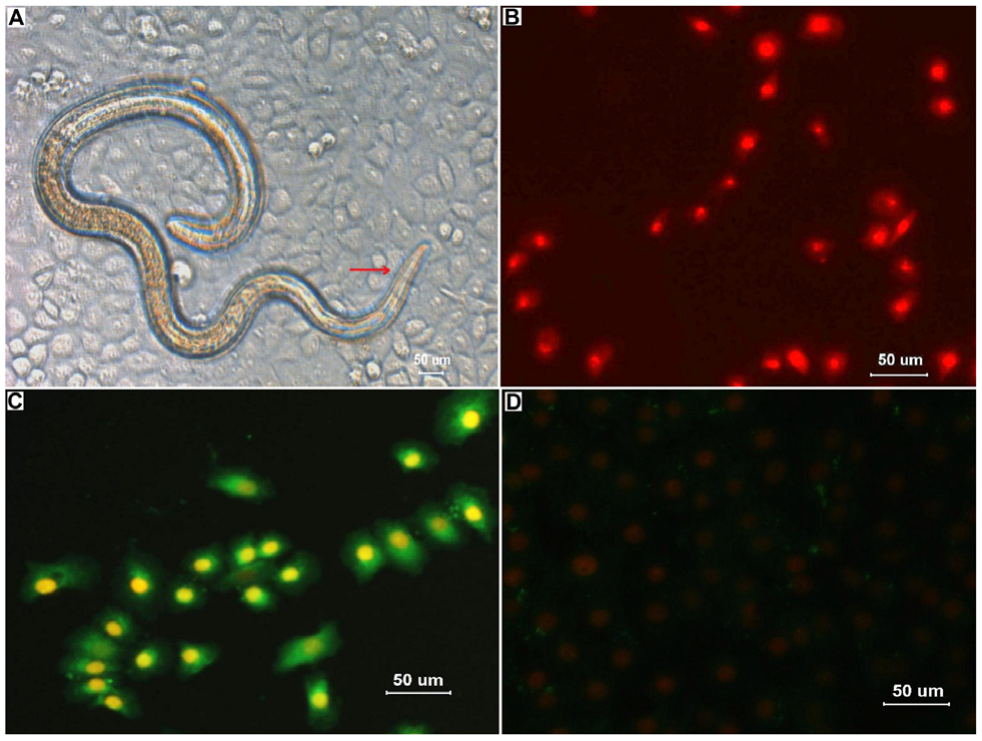
Observations on invasion of IECs by *Trichinella spiralis* infective larvae. (A) When *T. spiralis* infective larvae were inoculated onto monolayers of mouse IECs *in vitro*, the larvae invaded the cells, and their head and body resided in the cytoplasm of the syncytia composed of numerous IECs (showed as arrow). (B) After the removal of agarose, the cell monolayer was stained with propidium iodide (PI) and observed under a fluorescence microscope. Nuclei of the damaged cells were stained intensely and uniformly red, showing the serpentine trail left by the parasite. In contrast, nuclei of the live cells not invaded by the larvae were not stained. (C) After being stained with PI, the cell monolayer was fixed and reacted with rabbit immune sera against *T. spiralis* ES antigens and FITC-conjugated secondary antibody as described in Materials and Methods. Green fluorescence was found in the cytoplasm of the damaged cells. (D) No fluorescence was found in the cells which were not invaded by the non-activated larvae.

When IEC monolayers were overlaid with the activated larvae suspended in semisolid medium, the larvae browsed the IEC surfaces, quickly invaded the monolayers and migrated through contiguous cells (Figure 4A). The results of trypan blue staining showed that the infective larvae caused a significant amount of IEC damage. The mean area ([65±12]×10^3^ mm^2^) of damage to monolayer caused by activated larvae was significantly greater than that (0.2×10^3^ mm^2^) of damage to monolayer by non-activated larvae (*p*<0.01).

Trails of the damaged cells caused by the larvae that migrated through the monolayers were left. These trails were documented by nuclear staining with PI (Figure 4B). In addition, the dead cells were recognized by rabbit immune sera (Figure 4C). In contrast, when the monolayers were inoculated with the non-activated larvae, the larvae did not invade the cells and these cells were not recognized by rabbit immune sera (Figure 4D).

## Discussion

**Figure 5:**
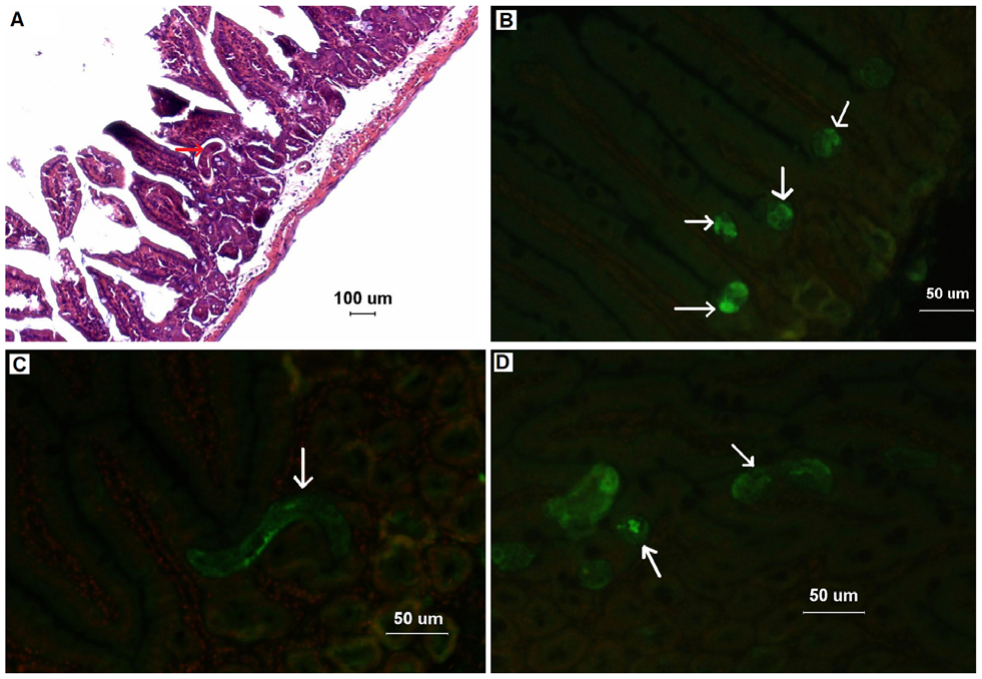
Hematoxylin-and-eosin (HE) staining and immunofluorescence test (IFT) on the small intestines of mice infected with *Trichinella spiralis*. (A) The Kunming mice were orally infected with 300 *Trichinella spiralis* muscle larvae. After 18 hours, they were killed by anaesthetic inhalation with isoflurane, and their small intestines were fixed in 10% formaldehyde solution. HE staining showed that the larvae located at the crypt-villus junction (showed as arrow). (B and C) Different sections of the larvae at the crypt-villus junction were recognized by sera of the rabbits infected with *T. spiralis*, and exhibited green fluorescence (showed as arrows). The mouse small intestines exhibited only background red fluorescence (propidium iodide). (D) Longitudinal sections of the larvae (showed as arrows) were found in intestinal crypts (green fluorescence).

IECs play a central role in absorptive and secretory functions, providing an effective barrier against the complex antigenic load of intestinal contents. However, the continuous and rapid renewal of IECs lies in a process involving cell generation and migration from the stem cell population located at the bottom of the crypts to the extrusion of the terminally differentiated cells at the tips of the villus [15]. Obviously, the isolated crypt cells with a high proliferation rate can be the origin of IECs *in vitro*. Otherwise, *T. spiralis* larvae and adults localize at the crypt-villus junction after infecting the host [5]. This conclusion was confirmed in our study by HE staining (Figure 5A) and IF test (Figure 5B, 5C and 5D) of the small intestines of mice infected with *T. spiralis*. Therefore, mouse intestinal crypts were isolated and cultured *in vitro* for harvesting IECs in this study. The monolayers of cultured IECs invaded by *T. spiralis* will provide the natural model of invasion to mimic the normal intestinal epithelium *in vivo*.

Indeed, the successful *in vitro* cultures of IEC from mouse fetal intestine in our study indicated that primary IECs could be obtained more easily from fetal tissue [16]. Compared with the tissues of adult and new-born mouse, fetal tissues were selected for the following three reasons. Firstly, fetal tissues can be obtained under sterile conditions. Secondly, fetal tissues seem to be more effective for the generation of epithelial cells because of their rapid metabolism and self-renewing rate [17]. Thirdly, the structure of the villus at E15–E16 develops rapidly and the epithelium changes from a stratified to a monolayer shape [18], [19]. Moreover, fetal cells in the small intestine might be abundant in dividing cells with potency to differentiate into mature IECs at E17–19 [16]. In previous studies, the primary IECs were isolated from the intestines by vigorous shaking or using dissociating solutions containing collagenase and/or other proteases [17], [20], [21]. In our study, four different protocols of enzymatic digestion were tested (trypsin, collagenase, thermolysin, and a combination of collagenase and hyaluronidase) to optimize epithelial cell isolation. We found that the combination of collagenase and hyaluronidase was more effective in the release of intestinal crypts and the isolation of purer viable IECs *in vitro*. Additionally, the intestine should be finely cut into pieces rather than mashed. During enzymatic digestion, overly strong shaking should be avoided. Our results indicated that the cultured cells retained the ability to proliferate continuously and express specific cytokeratins which are the markers of epithelial differentiation and the main structural proteins in epithelial cells.

Generally, the mechanism by which intestinal nematodes invade intestinal mucosa may involve both mechanical and chemical damage. For example, the hyaluronidase released by *Ancyclostoma caninum* and *Anisakis simplex* within the host small intestine may be related to degrade mucosa, invasion, and histolysis [22]. *Trichuris* has been reported to produce a pore-forming protein [23] and also employs a buccal stylet to mechanically pierce cells. As for the infective larvae of *T. spiralis*, they do not possess oral appendices or a spike [24], implying that invasion of intestinal epithelial cells by them may be not simply a result of mechanical penetration. Thus, there is an urgent need for the establishment of an *in vitro* model which provides a readily manipulated system to further investigate the invasive process and to study the cellular and molecular mechanisms of invasion by *T. spiralis*. In recent years, some studies have already established several invasion models by using tumor cell lines [7], [25]. In the present study, we established successfully a normal intestinal epithelial model of invasion *in vitro*. The cultured mouse IECs *in vitro* were susceptible to invasion by *T. spiralis* larvae. When the larvae were inoculated onto the monolayers, they penetrated and migrated through cells leaving serpentine trails of damaged cells which were observed under a fluorescence microscope. The results suggested that some ES antigens secreted by the larvae were left in the cytoplasm when they invaded IECs. These data will help us to screen the proteins related to invasion of small intestine by *T. spiralis* in further studies.

The pivotal indispensable requirements for invasion by *T. spiralis* are the physical support of agarose, the activation of the larvae, and epithelial cell monolayers [7], [26]. However, we also found that when the larvae were suspended in liquid medium and inoculated onto the monolayers, they used their heads to probe and poke at the surfaces of cells, and then penetrated the IECs, although only their heads were viewed in the cells under a light microscope. Certainly, no trails of damaged cells can be observed without agarose by providing a physical support for the worm.

In conclusion, we established an *in vitro* normal intestinal epithelial model for studying the invasion of IECs by *T. spiralis*. Our studies showed that primary mouse IECs have been successfully isolated and cultured from fetal mouse intestines. The cultured cells were found to retain the morphological and immunological characteristics of IECs, and they were susceptible to invasion by *T. spiralis*. This normal invasion model mimicking the natural environment *in vivo* provides a readily manipulated and controlled system to further investigation of the niche of *T. spiralis*, as well as the mechanisms of immune system-mediated disruption of the niche.
